# Lifestyle Changes during the SARS-CoV-2 Pandemic as Predictors of BMI Changes among Men and Women in Poland

**DOI:** 10.3390/nu15112427

**Published:** 2023-05-23

**Authors:** Izabela Bolesławska, Paweł Jagielski, Ewa Błaszczyk-Bębenek, Anna Jagielska, Juliusz Przysławski

**Affiliations:** 1 Department of Bromatology, Poznan University of Medical Sciences, 3 Rokietnicka Street, 60-806 Poznan, Poland; jprzysla@ump.edu.pl; 2 Department of Nutrition and Drug Research, Institute of Public Health, Faculty of Health Sciences, Jagiellonian University Medical College, 8 Skawińska Street, 31-066 Kraków, Poland; paweljan.jagielski@uj.edu.pl (P.J.); ewa.blaszczyk@uj.edu.pl (E.B.-B.); 3 Department of Social Medicine and Public Health, Medical University of Warsaw, 3 Oczki Street, 02-007 Warsaw, Poland; anna.jagielska@wum.edu.pl

**Keywords:** SARS-CoV-2, body mass index, social distancing, eating behavior, physical activity, sleep duration

## Abstract

Background: Social isolation during the SARS-CoV-2 pandemic affected people’s body weight, therefore, this study was designed to evaluate the association between lifestyle elements and the change in BMI during lockdown. Methods: This retrospective observational study involved 290 questionnaires completed by adult participants divided into three groups according to BMI change during isolation. The structured questionnaire included a general description of the study objective and collected data regarding sociodemographics, anthropometrics, physical activity, sedentary behaviour, sleep duration, and food intake pre- and during COVID-19 lockdown. Results: A decrease or increase in BMI was found in 23.6% and 47.8% of women and 18.5% and 42.6% of men, respectively. Among those who lost weight, 46.5% of women and 40% of men followed a diet of their own choice, 30.2% of women and 25% of men changed their product mix and reduced their intake, 40% of men stopped eating outside the home. An increase in BMI was associated with increased food intake (32.2% of women and 28.3% of men), increased sleep duration on weekdays (49.2% of women and 43.5% of men) and, in more than 50% of subjects, decreased physical activity. In women, increased BMI was associated with the highest frequency of snacking (*p* = 0.0003), the highest intake of sweets (*p* = 0.0021), and in men with the highest intake of alcohol (*p* = 0.0017). Conclusions: The observed changes in BMI during social isolation were the result of lifestyle modifications including dietary behaviour and differed by gender.

## 1. Introduction

The unprecedented social isolation in more than 100 years occurred due to restrictions imposed worldwide to protect against the spread of the SARS-CoV-2 in 2020 profoundly disrupted daily life, leading to changes in the habitual functioning of populations, and affected mental and physical health [1]. The main restrictions were on maintaining social distance and maintaining hygiene. The ban on moving around the country and abroad, changing to a remote form of work or study, and the recommendation not to leave one’s place of residence and other non-standard arrangements important for staying safe during this period [2,3,4,5], promoted staying in the same place and resulted in a significant reduction in physical activity [6,7,8,9,10,11,12], an increase in time spent sitting [6,10], especially screen time [12,13], and increased [14,15] or decreased sleep [10,15], as well as deteriorated sleep quality [8,16,17,18]. Reduction in shop operations and temporary shortages of groceries [6,19] also affected diets [20,21]. Changes in shopping habits, such as a reduction in the frequency of in-store purchases and an increased interest in online grocery shopping, were also observed. There was an increase in the frequency of buying frozen products and foods with a long shelf life and stockpiling at home. The frequency of consumption of dairy, cereals, fat, vegetables, sweets, and alcohol increased [19]. In addition, the real threat of COVID-19 infection during this period induced high levels of psychological stress, fear, and anxiety [22,23], further influencing the disruption of previous behavior [24].

However, interestingly, the lockdown caused by the COVID-19 pandemic has not only led to a consolidation of unfavourable eating habits but also a correction of previous inappropriate eating patterns and behaviors [25]. COVID-19-related limitations differentially affected depressive and anxiety symptoms, sleep disturbances, physical activity habits, diet, and sedentary time according to gender [16,26,27].

Studies in humans and animal models suggest that being in identical conditions of social isolation can lead to both weight gain and an inflammatory phenotype in adipocyte adipose tissue and weight loss [28,29,30] and, in vulnerable groups (adolescents, and people with post-traumatic stress), an increase in the adoption of unhealthy weight-control practices [31,32]. Resulting excess or deficiency of body weight is dangerous for clinical COVID-19 outcomes, increasing the risk of severe disease course, hospitalisation, and death [33,34,35,36,37,38,39,40]. With the metabolic tissue burden induced by weight gain and adipose tissue dysfunction exacerbating the inflammation caused by SARS-CoV-2 virus contributing to systemic chronic inflammation of low-grade malignancy, overexpression of ACE2 receptors in obesity may support infection and act as a reservoir for the virus [41,42,43] and comorbidities (including atherogenic dyslipidaemia, hypertension, cardiovascular disease, and type 2 diabetes) further impair organ and system function, thereby worsening the course of infection [42,44]. Malnourished patients also had greater systemic inflammation and longer COVID-19 duration [45] and were more likely to require mechanical ventilation or die [46]. This was due to the lipodystrophy that occurs in malnutrition [34], altered immunometabolism and an impaired protective response [47,48,49], and reduced protein stores [50].

Obesity was a huge global problem before the emergence of the SARS-CoV-2 pandemic [51]. Pandemic has exacerbated obesity risk factors, increased the number of people with obesity [20,52,53,54], and will worsen obesity rates in the near future [55,56,57]. On the other hand, the acute stress associated with COVID-19 had anorexigenic effects in some individuals, leading to reduced food intake and malnutrition [58,59].

Habit in humans is a key determinant of behaviour maintenance [60]. People tend to repeat the same behaviours in similar, repetitive contexts [61]. The constraints associated with the COVID-19 pandemic disrupted almost every aspect of life, including existing habits related to work, study, physical activity, and access to educational, cultural, medical, and catering facilities, by causing not only transient behavioural and dietary changes, but a permanent transformation related to changes in consumer choices [62], social habits [63], physical activity [64], increased psychological stress, and sleep disturbance [65]. In many individuals, this has influenced changes in BMI.

The aim of this study was to identify which lifestyle factors (dietary adherence, eating habits, physical activity, sedentary behaviour, and sleep duration) and sociodemographic characteristics influenced, and how they influenced, the change in BMI during COVID-19 closure. This will allow these factors and characteristics to be better controlled and enable the implementation of effective, gender-specific interventions to reduce the serious public health crisis associated with the increasing prevalence of obesity or malnutrition by shaping favourable health, dietary, physical activity habits, and choices. Well-formed habitual behaviours can be more easily reactivated in future outbreaks requiring isolation. Identifying the causes of changes in BMI will also help address the distant consequences of isolation caused by the SARS-CoV-2 pandemic.

## 2. Materials and Methods

### 2.1. Study Design

To assess lifestyle changes during the SARS-CoV-2 pandemic and their impacts on increasing or decreasing BMI, a retrospective observational study was conducted from 29 April to 19 May 2020 in healthy adults in Poland who did not become ill with COVID-19 before or during the study. The survey questionnaire included questions on the COVID-19 lockdown period, which covered the period of the introduced epidemic state from 20 March to 30 May 2020 and the period six months before the start of the COVID-19 lockdown (before the lockdown). For weight and height, we asked for measurements from the last three months before the start of the COVID-19 lockdown. The survey was completed during the COVID-19 lockdown. Inclusion criteria were age 18 to 65 years and the ability to give informed consent to participate in the study. Subjects under 18 years of age and over 65 years of age, those with COVID-19, those on a therapeutic or alternative diet, and pregnant women were excluded. The survey questionnaire was posted and made available on Google’s online survey platform. The link to the electronic survey was disseminated through various methods to reach a wide range of respondents: posting on the official profile of the Institute of Public Health, on the accounts of the researchers taking part in the survey, via email, and sharing on social media sites Facebook, WhatsApp, and Instagram.

Study participation was voluntary and participants could withdraw from the study at any stage. Participants were informed that anonymity and the purposes and scope of the study would be maintained, and they provided informed consent to participate. The study was conducted in accordance with the principles of medical research ethics contained in the Declaration of Helsinki and was approved by the local ethics committee of the Warsaw Medical University (no. AKBE/122/2020).

**Figure 1:**
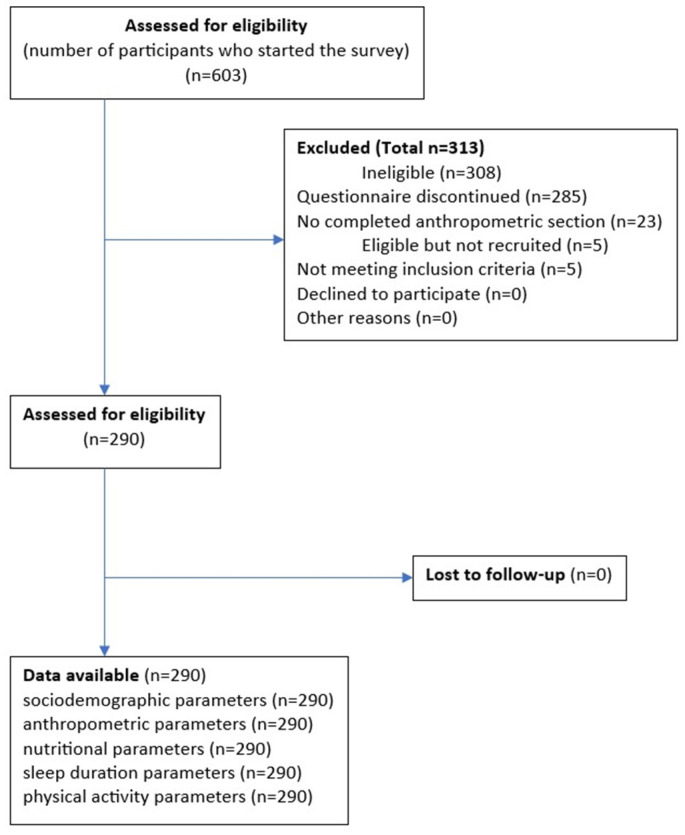
The flow of participants through the study based on the STROBE guidelines [66].

The study included 290 participants from 15 provinces in Poland, of whom, 62.8% were women and 37.2% were men. The response rate was 48.1% (number of surveys completed and sent divided by number of surveys started × 100%). The questionnaire was started by 603 participants (they answered the first question). Of these, 285 participants did not complete the questionnaire, 23 did not complete the anthropometric section, and 5 did not qualify due to not meeting the inclusion criteria (pregnancy, and age). Complete questionnaires were obtained from 290 participants for analysis and the flow of participants is shown based on the STROBE guidelines [66] in Figure 1.

The minimum sample size was calculated before the start of the study using Surveymonkey.com sample-size-calculator [67], assuming 23,029,800 adults in Poland in 2019 [68]. With an assumed 95% confidence level (α = 0.05) and a standard error of estimate of 6%, the minimum sample size was 267 persons.

### 2.2. Measurements

#### 2.2.1. Survey Questionnaire

The research tool was a web-based questionnaire containing 96 questions designed to collect information regarding the respondents’ sociodemographic data (age, gender, employment, education, and type of work), selected dietary behaviour, sleep duration, sedentary behaviour, and physical activity, as well as weight measurements before and during the SARS-CoV-2 pandemic. The questions were developed based on the validated and recommended standards for research in the Polish population Questionnaire of Eating Habits and Food Beliefs for Persons Aged 15–65 Years (KomPAN) developed by the Behavioral Nutrition Team of the Committee on Human Nutrition at the Polish Academy of Sciences [69]. Completed questionnaires were uploaded to the Jagiellonian University survey platform, while the final database was downloaded as a Microsoft Excel spreadsheet.

Questions on age, body height, gender, education, and place of residence were asked once. The questionnaire also asked participants about their employment, with the options to answer: (a) I am retired, or on a pension, (b) I am on maternity leave, unemployed, or other, (c) I have a part-time job, (d) I have a permanent job, and (e) I am in education or studying. The section regarding dietary habits included questions on the use of diet with possible answers: (a) no, (b) yes, on doctor’s orders for health reasons, and (c) yes, of my own choice; the number of meals eaten per day with the possibility of recording the number of meals consumed; frequency of snacking with answers and scores: (a) never—1, (b) 1–3 times a month—2, (c) once a week—3, (d) several times a week—4, (e) once a day—5, and (f) several times a day—6; frequency of consumption of fast food, fried foods, sweets, alcohol, sweetened beverages, vegetables, and fruit with the options to answer: (a) never, (b) 1–3 times a month, (c) once a week, (d) several times a week, (e) once a day, and (f) several times a day, and frequency of eating out (including home delivery), e.g., in bars, restaurants, cafés, and canteens, with the options to answer: (a) never, (b) 1–3 times a month, (c) once a week, (d) several times a week, and (e) once a day.

There was also a question not included in the KomPAN questionnaire regarding the change in the form of work during isolation caused by COVID-19 compared to before with the options to answer: (a) yes, I work as before, (b) yes, I work remotely from home, (c) no, (d) no, I am on unpaid/paid leave looking after a child/children under 8 years old, and (e) not applicable, and changes in diet due to social isolation with possible answers: (a) no change, (b) eat the same products in larger quantities, (c) eat the same products in smaller quantities, (d) changed the range of products without changing the quantities, (e) changed the range of products and increased the quantities, and (f) changed the range of products and decreased the quantities.

The questionnaire also included questions on the number of hours of sleep on weekdays and weekends, with responses and scores: (a) 6 h or less/day—1, (b) more than 6 but less than 9 h/day—2, and (c) 9 h or more/day; sedentary behavior: (a) <2 h, (b) 2–4 h, (c) 4–6 h, (d) 6–8 h, (e) 8–10 h, and (f) >than 10 h; and a question on physical activity during work or school time, with three possible levels with scores—low: more than 70% of the time is sitting—1, moderate: about 50% of the time is sitting and 50% activity—2, and high: about 70% of the time is high-intensity activity or physical work—3; and physical activity in leisure time, with responses and scores, respectively—low: mainly sedentary lifestyle, watching TV, reading newspapers/books, light housework, or walking for 1–2 h/week—1, moderate: walking, cycling, exercising, gardening, or other light physical activity for 2–3 h/week—2, and high: cycling, running, gardening, and other sports/recreational activities requiring physical activity for more than 3 h/week—3. Respondents were asked the same questions twice regarding the time before and during lockdown due to the SARS-CoV-2 pandemic.

The questionnaire was supplemented with questions on anthropometric parameters.

#### 2.2.2. Body Weight and BMI

Participants reported their weight (kg) measurements before and during the lockdown based on measurements taken on household scales. Body height was given only once, based on measurements with a height meter. Based on the declared measurements, body mass index (BMI—kg/m^2^) as the quotient of the weight of a body to its height in meters squared was calculated for each participant at two time points. The interpretation of nutritional status based on BMI was based on the World Health Organization (WHO) guidelines for adults [70].

In order to separate the study participants into groups with an increase/decrease or no change in BMI, we relied on the following scheme: a BMI was calculated for each respondent on the basis of body weight and height obtained. Those for whom the difference between BMI during lockdown and before the SARS-CoV-2 pandemic was <0.00 were classified in the group with a decrease in BMI, and those for whom the difference in BMI was >0.00 were classified in the group of people who had gained weight. If there was no change in BMI—they were assigned to the group with no change. For men and women, we made an identical allocation and it was as follows: women and men who experienced a decrease in weight and BMI (men 18.5% of the sample group of men; weight—2.20 kg, BMI—0. 70 kg/m^2^; and women 23.6% of the surveyed group of women; weight—2.00 kg, BMI—0.71 kg/m^2^), weight gain and BMI (men 42.6% of the sample group of men; weight 2.00 kg, BMI 0.66 kg/m^2^; and women 47.8% of the surveyed group of women; weight 2.00 kg; BMI 0.74 kg/m^2^), or no change in weight and BMI (28.6% of women and 38.9% of men, *p* < 0.05). Based on the subgroups thus formed, we analyzed the effect of individual factors on BMI during the COVID-19 lockdown. For physical activity, the subgroups with increase/decrease/no change in BMI were separated within the entire study group with no division between men and women.

### 2.3. Statistical Analysis

Age and anthropometric data are presented as mean ±SD and other categorical variables as percentages. The subjects were divided into three groups: those whose BMI decreased, those with no change in BMI, and a third group, those whose BMI increased. The results were presented at the same time by gender. The McNemar–Bowker test was used to check the differences between categorical variables before and during isolation. The Kruskal–Wallis test or Chi-squared test were used to check for differences between variables in the three groups dependent on changes in BMI. In addition, changes in physical activity among study participants before and during isolation were illustrated using Sankey charts. Data analysis was performed with PS IMAGO PRO 6 (IBM SPSS Statistics 26) assuming a statistical significance of  <0.05.

## 3. Results

A decrease in BMI was reported by 23.6% of women and 18.5% of men, while 47.8% of women and 42.6% of men reported an increase. The subgroups of women who had a decrease/increase or no change in BMI differed significantly in terms of weight before isolation due to the SARS-CoV-2 pandemic (*p* = 0.0072), age (*p* = 0.0004), and body height (*p* = 0.0252), while there were no differences in terms of place of residence, education, and employment. The mean BMI in these groups of women was also similar both before and during lockdown. The group of women who had a reduced BMI during isolation had the highest mean body weight before the SARS-CoV-2 pandemic. Among these women, almost half followed a diet of their own choice (46.5%), which may explain the observed weight loss. This was also the youngest group of women (mean age 34.5 ± 10.0 years).

Men, regardless of BMI changes, were characterised by similar age, place of residence, education, and type of work as well as body weight and BMI before and BMI during lockdown (*p* > 0.05; Table 1). In the group of men who reported a reduction in BMI, a large proportion (40%) also followed a diet of their own choice during lockdown.

The change in the structure and quantity of dietary intake during the isolation caused by the SARS-CoV-2 pandemic played a significant role (*p* < 0.0001; Table 2), with the highest proportion of women (32.2%) and men (28.3%) who reported an increase in BMI during the lockdown consuming a greater quantity of the same products. Most women (30.2%) in the subgroup with an observed reduction in BMI changed their product mix and reduced their intake. In contrast, 35% of men who reported a reduced BMI ate the same foods in the same quantity but one quarter changed their product mix and reduced their product intake.

The number of meals consumed per day did not differ between the subgroups of men and women during lockdown. The significant dietary factors that influenced the women’s increased BMI were the highest frequency of snacking (*p* = 0.0003) and the weight of sweets consumed per day (*p* = 0.0021), whereas it was the quantity of alcoholic beverage consumption (*p* = 0.0017) for men (Table 3).

No significant difference was observed in the frequency of meals eaten outside the home before and during lockdown (*p* < 0.05) between the subgroups of women (Table S1). However, among men, the frequency of eating out differed significantly before isolation (*p* = 0.0454) but no longer during isolation (*p* = 0.2636). Of the men who reported a reduction in BMI, up to 40% stopped consuming food outside the home.

Interestingly, only 14% to 16.3% of women worked as before, while 44.8–48.8% changed to working remotely and 7–8% of the women looked after children under the age of 8. Between 40 and 44.5% of the men changed to working remotely, with 30–38.1% of them working during the lockdown as before. However, changes in work form during lockdown were similar in all subgroups of women and men (*p* > 0.05; Figure S1).

Increased sleep duration on weekdays was reported by almost half of the women and 43.5% of the men in whom BMI increased during lockdown, as well as in 40% of men in whom BMI decreased (*p* < 0.05; Table 4). During lockdown, sleep duration lengthened in approximately 20% of women with an observed increase or decrease in BMI but sleep duration decreased (*p* = 0.0017) in a similar number of women with an observed decrease in BMI. There were no significant changes in sleep length on weekends between the subgroups of men (*p* > 0.05).

**Figure 2:**
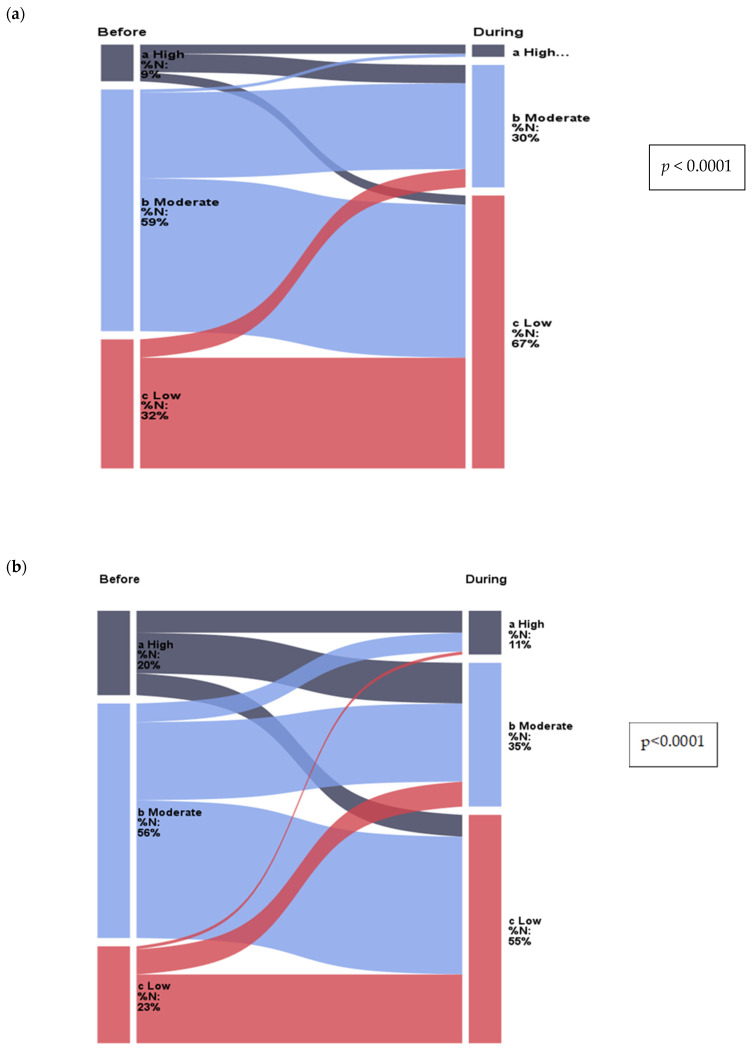
Change in physical activity in individuals who gained weight (**a**) during work: before the introduction of isolation, 9% of participants had high physical activity and 32% had low physical activity, while during the lockdown, 3% of participants had high physical activity and 67% had low physical activity; (**b**) at leisure time: before isolation, 20% of participants had high physical activity and 23% had low physical activity, while during lockdown, 11% of participants had high physical activity and 55% had low physical activity.

In the group of subjects with an increase in BMI, more than 50% decreased their physical activity during work and leisure time (*p* < 0.0001, and *p* < 0.0001; Figure 2). The same was true for the group of subjects whose BMI did not change (*p* = 0.0198, and *p* = 0.0257). Only subjects who reported a decrease in BMI during lockdown maintained a similar amount of physical activity during work (*p* = 0.3978) and leisure time compared to the previous period (*p* = 0.3441; Figures S2 and S3).

The question on physical activity during work or school time had three possible levels with scores—a—low: more than 70% of the time is sitting—1; b—moderate: about 50% of the time is sitting and 50% activity—2; c—high: about 70% of the time is high-intensity activity or physical work—3; and in leisure time, with responses and scores, respectively—low: mainly sedentary lifestyle.

While time spent in front of a monitor increased during the SARS-CoV-2 pandemic in all study groups, there were no significant differences between the male and female subgroups both before and during isolation (*p* > 0.05, Table 5).

## 4. Discussion

Living in social isolation due to the SARS-CoV-2 epidemic was associated with several lifestyle changes which have influenced changes in BMI. In the present study of a Polish population, weight gain during isolation was reported by almost half of women and more than 40% of men, similar to a study conducted in Italy (48.6%) [9]. The percentage of subjects who gained weight exceeded the number reported in the USA (40%) [71], China (30.6%) [72], and France (35%) [6], as well as in Poland (affecting 30% of subjects) [73]. In contrast, weight reduction was reported by almost 20% of women and men, similar to other studies from this period [73].

There were no differences between the subgroups of women and men regarding their place of residence, education, or type of employment. Interestingly, men were also characterized by similar age regardless of BMI changes. Several studies have shown that older age is associated with weight gain during the COVID-19 lockdown [73]. The oldest age group of women in our study (mean age 45.0 years) maintained the same BMI, while changes in BMI were observed in the younger age groups—a decrease in BMI (mean 34.5 years) or an increase in BMI (40.7 years).

Energy deficit is the most important factor influencing weight loss and BMI reduction [74], hence, the use of dieting by almost half of the women and 40% of the men who achieved weight loss was the major reason for this. However, weight management is influenced by the type of food in addition to the amount consumed [74]. In the group of people who experienced a reduction in BMI, the largest proportion of women and one quarter of men changed their product mix and reduced their food intake. This is a very positive development, as the introduction of energy restriction and, at the same time, good-quality food not only lead to a reduction in body weight but is also fundamental to the effective impact of the diet on health [75]. In contrast, the largest proportion of women and men who experienced an increase in BMI during the isolation caused by the SARS-CoV-2 pandemic increased their consumption of both previously consumed foods and a new range of foods. A similar relationship was observed in a study by Górnicka [76] conducted in Poland as well as other studies from this period [77].

For effective weight maintenance or weight reduction, it is also important to maintain appropriate meal times, eat meals with higher energy value earlier in the day, and maintain consistent periods of overnight fasting. In several cross-sectional studies in free-living populations, a higher eating frequency was associated with a lower risk of obesity [78]. In the present study, the number of meals consumed per day did not differ in the subgroups of men and women with a change in BMI. However, an increase in BMI during COVID-19 isolation in women was associated with the highest frequency of snacking and the weight of sweets consumed per day. Increased snack intake during COVID-19-induced isolation was quite common and observed in both children [79] and adults [80], especially among women [73,81]. Similarly, excessive consumption of sweets was observed during COVID-19 isolation in other groups of women [80,81].

Snack consumption does not necessarily result in increased body weight. The inclusion of 1–2 snacks per day alleviates potential gastrointestinal and metabolic overload caused by fewer heavier meals and may contribute to meeting recommendations for food groups (e.g., fruit, and dairy) and nutrients, such as fibre and vitamins [82]. However, if snacks are habitually consumed without energy reduction they can cause a positive energy balance. Therefore, there is a consensus that nutrient-poor and high-energy snacks may contribute to higher energy intake and cause weight gain in adult populations [83]. The context in which adults consume snacks, e.g., eating alone, away from home or work, late in the day, and in front of the TV or computer, is also relevant to this behaviour [83] and, in the case of isolation caused by the introduction of restrictions to reduce the spread of the SARS-CoV-2 virus, excessive sweets consumption was associated with boredom and anxiety resulting from being confined to the home [81].

The association between high sugar intake and weight gain is not in doubt [84]. Also, the consumption of foods and beverages with sucrose and fructose is a risk factor for obesity [85]. This is all the more so because, in addition to increasing the dietary caloric content, such foods can cause neuroadaptations in the reward system that separate eating behaviour from caloric needs and lead to compulsive overeating [86].

The increased consumption of alcoholic beverages observed in a group of men with increased BMI was also an independent predictor of weight gain in studies by other authors dating back to the COVID-19 lockdown [87] and was additionally associated with increased depression, anxiety, and stress [88]. Alcohol drinkers are less likely to positively reframe (find something positive in the COVID-19 pandemic situation) and are less mentally able to cope with the situation [89]. According to the present study, the lowest alcohol consumption in men during the lockdown caused by the SARS-CoV-2 pandemic was also associated with a reduction in BMI. In the subgroup of men who reported a 40% reduction in BMI, the proportion who ate out also decreased. Eating in restaurants is associated with higher intakes of energy, total and saturated fat, sugar and sodium, and beer and other alcohol, and lower intakes of fibre, dairy, fruit, vegetables, and micronutrients [90]. A study by Bhutani et al. [91] found that an increase in fast food and sit-down restaurant food intake by one meal/week was associated with an increase in BMI of 0.8 and 0.6 kg/m^2^, respectively. Thus, a reduced intake of such meals significantly contributes to weight reduction and lower BMI in this subgroup of men.

A change in the type of work from stationary to remote also lead to weight gain during the lockdown caused by the SARS-CoV-2 pandemic [92]. According to the present study, more than 40% of women and men changed to working remotely, however, the changes in work form during the lockdown were similar in all subgroups of women and men. Hence, it appears that the change in work form was not the cause of the changes in body weight and BMI in this group of subjects.

The observations on sleep duration were interesting. Although the causal relationship between sleep duration and obesity is not clear [93,94], epidemiological studies have established short sleep duration as a risk factor for the development of obesity during the COVID-19 lockdown [77,93,95]. Short sleep duration may be associated with an increase in the orexigenic hormone ghrelin, which stimulates hunger, and a decrease in the satiety hormone leptin, resulting in increased food intake to combat fatigue or stress and reducing the effectiveness of dietary adherence [96]. In turn, increasing sleep duration and correcting sleep disturbances post-balances appetite-regulating hormones, increases glucose tolerance, and lowers cortisol levels [97]. The highest rates of obesity were observed in people sleeping <7 h per day [98,99]. In our study, prolonged sleep duration on weekdays occurred in 40% of men who reported a decrease in BMI during lockdown. However, a significant percentage of men and women who reported an increase in BMI slept longer on working days, which is very interesting in the context of previous literature findings.

The lockdown to curb the spread of the SARS-CoV-2 pandemic also resulted in decreased physical activity and increased sedentary behaviour [6,26], which adversely affected immune function and increased the risk of illness and hospitalisation associated with viral infection [100,101]. It also rendered it more difficult to maintain normal body weight and may have caused weight gain in a large proportion of individuals [102,103]. The present study confirmed the association between decreased physical activity during work and leisure time, and an increase in BMI during lockdown. More than 50% of the subjects in whom an increase in BMI occurred decreased physical activity. Only those who reported a decrease in BMI during lockdown maintained a similar amount of physical activity during work and leisure time compared to the previous period.

Several studies suggest that negative changes in physical activity have a particularly negative impact among those with higher BMI [11,55,73], as confirmed in the men in the present study. Our study did not include an assessment of mood and its impact on weight changes but given that work-related stress among men with a high baseline BMI is one of the main drivers of further weight gain [104], it is conceivable that it may have been responsible for the increase in BMI. Interestingly, in our study, the women with the highest BMI before lockdown had a decrease in BMI with no change in physical activity. This may have been due to both the need to combine work, household duties, and childcare during the lockdown as well as the introduction of beneficial physical activity behaviours that were observed during lockdown in, for example, women in the United Kingdom, Ireland, New Zealand, and Australia [26].

Increased sedentary behaviour may impede the maintenance of normal body weight and BMI during lockdown [6,26]. However, there were no significant differences in time spent in front of a TV/monitor and increase/decrease in BMI in either the female or male subgroups in the present study.

In this study, we attempted to capture as accurately as possible the potential lifestyle factors responsible for the change in BMI in men and women during the SARS-CoV-2 lockdown. The speed with which the surveys were implemented ensured that the population response to the relevant government-mandated mitigation strategies was captured at similar levels. Despite the large amount of data, we are aware of some study limitations that need to be highlighted, particularly in terms of survey methodology. First, this was a retrospective study assessing lifestyle behaviours before the COVID-19 lockdown. The study was conducted at the start of the pandemic. In addition, the change in body weight was self-reported and not measured by the researchers which may reduce the sensitivity of the study. Also, whether participants were infected with COVID-19 was not assessed, and, therefore, data on a possible bidirectional effect of weight change with COVID-19 infection could not be obtained. The questionnaire was completed online and not in the presence of a dietician due to COVID-19 restrictions so there may have been measurement errors, especially for anthropometric measurements. Finally, the completion rate of the questionnaire was low, possibly because the respondents could halt the survey at any time and/or not answer all questions or complete all sections, especially regarding the anthropometric parameters. However, although this may introduce significant errors when interpreting the results, many estimates based on telephone surveys with fairly low response rates overlap with estimates from surveys with higher response rates [105,106]. Also, the electronic surveys tend to be completed by respondents with higher education, probably due to greater accessibility to computers and better-quality internet connections, thereby limiting the representativeness of the group in relation to Poles in general. The questionnaire used, despite some shortcomings, was the only possible solution during the COVID-19 pandemic.

## 5. Conclusions

The very high proportion of people who experienced a change in BMI during the lockdown caused by the SARS-CoV-2 pandemic indicates an urgent need to identify the factors responsible for these changes. The present study provides insight into the impact of the lockdown caused by the SARS-CoV-2 pandemic on selected lifestyle components responsible for the increase or decrease in BMI according to gender. All the more so, as the impact of the lockdown caused by the SARS-CoV-2 pandemic on BMI is observed long after its end.

The increase in BMI is associated with increased food intake and sleep duration, decreased physical activity, and increased frequency of snacking and number of sweets consumed by women and alcoholic beverages by men, while a decrease in BMI with the range of products consumed and reduced intake and, in men, additionally a reduction in eating out and low alcohol intake will allow effective gender-specific interventions to be achieved in the future. Increasing physical activity by promoting exercise that can be done at home and improving sleep duration should be considered for future risks. Strategies to achieve and maintain a healthy body weight and BMI should be prioritised at the population level, which could help to reduce the burden of COVID-19, as well as potential future pandemics and other reasons requiring isolation, however, taking into account complex gender-dependent factors.

## Figures and Tables

**Table 1:** General characteristics of the subgroups of men and women with a decrease, no change, or increase in BMI during lockdown.

Variables	Changes in BMI during Pandemic							
	Women				Men			
	Decrease *n* = 43 (23.6%)	No Change *n* = 52 (28.6%)	Increase *n* = 87 (47.8%)	*p*	Decrease *n* = 20 (18.5%)	No Change *n* = 42 (38.9%)	Increase *n* = 46 (42.6%)	*p*
Age (years)	x ± SD	x ± SD	x ± SD		x ± SD	x ± SD	x ± SD	
	34.5 ± 10.0	45.0 ± 12.4	40.7 ± 14.0	0.0004 *	42.3 ± 14.3	44.0 ± 12.1	40.0 ± 11.1	0.2563 *
Anthropometric parameters								
	x ± SD	x ± SD	x ± SD		x ± SD	x ± SD	x ± SD	
Body weight before (kg)	71.5 ± 14.1	63.5 ± 10.1	64.8 ± 11.7	0.0072 *	83.1 ± 9.22	83.8 ± 14.8	89.3 ± 17.9	0.3496 *
Body weight during (kg)	68.9 ± 13.7	63.5 ± 10.1	67.3 ± 12.1	0.0853 *	80.2 ± 8.46	83.8 ± 14.8	92.0 ± 18.7	0.0153 *
BMI before (kg/m^2^)	25.5 ± 4.73	23.6 ± 3.58	23.6 ± 3.88	0.0571 *	26.2 ± 3.61	26.1 ± 4.25	27.0 ± 84.69	0.6822 *
BMI during (kg/m^2^)	24.6 ± 4.67	23.6 ± 3.58	24.5 ± 3.97	0.3420 *	25.3 ± 3.45	26.0 ± 64.25	27.8 ± 94.90	0.0557 *
Body height (cm)	167 ± 4.92	164 ± 5.48	165 ± 5.86	0.0252 *	178 ± 7.20	179 ± 7.27	181 ± 7.62	0.1994 *
Sociodemographic parameters								
	%—percentage of respondents			*p*	%—percentage of respondents			*p*
Education (%)								
Occupational	2.3	2.0	2.3	0.420 **	5.0	4.8	4.4	0.961 **
Secondary	18.6	14.3	24.1		20.0	21.4	17.8	
Higher	79.1	83.7	73.6		75.0	73.8	77.8	
Place of residence (%)								
Village	18.6	20.0	31.0	0.210 **	25.0	21.4	13.3	0.227 **
City < 20 thousand residents	7.0	14.0	10.3		15.0	11.9	2.2	
City of 20–100 thousand residents	9.3	20.0	16.1		15.0	19.0	13.3	
City > 100 thousand residents	65.1	46.0	42.5		45.0	47.6	71.1	
Type of work before isolation (%)								
Retired/pensioner	2.3	14.0	9.2	0.074 **	15.0	11.9	6.7	0.499 **
Parental leave/ unemployed	2.3	6.0	5.7		0.00	0.00	0.00	
Casual work	14.0	2.0	6.9		10.0	4.8	4.4	
Permanent employment	72.1	76.0	64.4		65.0	81.0	86.7	
Student	9.3	2.0	13.8		10.0	2.4	2.2	
Use of diet during isolation (%)								
No	46.5	67.3	78.2	0.002 **	50.0	81.0	78.3	0.038 **
Yes, for health reasons	7.0	11.5	5.7		10.0	4.8	10.9	
Yes, by personal choice	46.5	21.2	16.1		40.0	14.3	10.9	

x—mean, SD—standard deviation, %—percentage of respondents, *—Kruskal–Wallis test, **—Chi^2^ test.

**Table 2:** Change in dietary intake structure and quantity of foods in the subgroups of men and women reporting a decrease, no change, or an increase in body weight during lockdown.

Variables	Change in Structure and Quantity of Product Consumption during Lockdown (%)							
	Women				Men			
	Decrease *n* = 43	No Change *n* = 52	Increase *n* = 87	Chi^2^ Test *p*	Decrease *n* = 20	No Change *n* = 42	Increase *n* = 46	Chi^2^ Test *p*
	%—Percentage of Respondents				%—Percentage of Respondents			
Same food products and in the same quantity	18.6	42.3	28.7	<0.0001	35.0	59.5	13.0	<0.0001
Same products in greater quantities	7.0	9.6	32.2		5.0	9.5	28.3	
Same products in a smaller quantity	18.6	9.6	2.3		15.0	4.8	17.4	
Change in product mix with no change in quantity	16.3	15.4	17.2		0.0	11.9	19.6	
Change in product mix and reduction in quantity	30.2	9.6	3.4		25.0	2.4	2.2	
Change in product mix and increase in quantity	9.3	13.5	16.1		20.0	11.9	19.6	

**Table 3:** Dietary factors associated with BMI changes during isolation due to the SARS-CoV-2 pandemic in the female and male subgroups.

Variables	Changes in Dietary Factors during Lockdown							
	Women				Men			
	Decrease *n* = 43	No Change *n* = 52	Increase *n* = 87	Kruskal–Wallis Test *p*	Decrease *n* = 20	No Change *n* = 42	Increase *n* = 46	Kruskal–Wallis Test *p*
	x ± SD	x ± SD	x ± SD		x ± SD	x ± SD	x ± SD	
Number of meals/day	4.02 ± 0.91	3.92 ± 0.90	4.07 ± 0.83	0.6636	3.80 ± 0.83	3.79 ± 0.84	4.02 ± 1.02	0.2806
Frequency of snacking	3.91 ± 1.39	3.90 ± 1.68	4.79 ± 1.35	0.0003	3.80 ± 1.74	4.52 ± 1.38	4.52 ± 1.41	0.2216
Fast food (g/day)	5.30 ± 7.17	7.88 ± 16.5	12.6 ± 27.0	0.3021	13.0 ± 22.8	11.0 ± 17.5	22.2 ± 53.2	0.5811
Fried foods (g/day)	31.1 ± 50.5	26.9 ± 28.3	37.1 ± 37.3	0.0898	36.4 ± 32.6	48.0 ± 61.6	55.6 ± 63.2	0.5309
Sweets (g/day)	41.7 ± 68.6	26.1 ± 31.4	70.9 ± 95.9	0.0021	32.0 ± 65.7	26.2 ± 29.9	53.0 ± 89.3	0.2555
Alcohol (g/day)	28.2 ± 45.0	28.2 ± 47.8	28.8 ± 44.7	0.3918	21.4 ± 32.4	61.9 ± 130	106 ± 146	0.0017
Sweetened beverages (g/day)	21.2 ± 64.5	26.9 ± 68.4	34.7 ± 99.0	0.6726	60.2 ± 106	62.6 ± 172	100 ± 182	0.3554
Vegetables (g/day)	282 ± 247	230 ± 238	214 ± 208	0.2310	102 ± 99.2	110 ± 125	156 ± 184	0.3912
Fruit (g/day)	141 ± 134	178 ± 223	183 ± 186	0.4533	102 ± 94.4	95.3 ± 85.3	122 ± 151	0.9504

Accepted score for frequency of snacking—never—1, 1–3 times a month—2, once a week—3, several times a week—4, once a day—5, and several times a day—6; x—mean, and SD—standard deviation.

**Table 4:** Changes in sleep during lockdown in the male and female subgroups.

Variables	Changes in Sleep Duration during the Pandemic							
	Women				Men			
	Decrease *n* = 43	No Change *n* = 52	Increase *n* = 87	Chi^2^ Test *p*	Decrease *n* = 20	No Change *n* = 42	Increase *n* = 46	Chi^2^ Test *p*
	%—Percentage of Respondents				%—Percentage of Respondents			
Sleep time	Weekdays (%)							
decreased	14.0	9.6	4.6	0.0378	5.0	0	4.3	0.0278
no change	58.1	61.5	46.0		55.0	83.3	52.2	
increased	27.9	28.8	49.4		40.0	16.7	43.5	
Sleep time	Weekends (%)							
decreased	20.9	5.8	2.3	0.0017	5.0	0	8.7	0.1866
no change	60.5	82.7	74.7		70.0	83.3	63.0	
increased	18.6	11.5	23.0		25.0	16.7	28.3	

**Table 5:** Changes in time spent watching TV or in front of the computer in the subgroups of men and women during lockdown.

Variables	Changes in Time Spent Watching TV or in Front of the Computer							
	Women				Men			
	Decrease *n* = 43	No Change *n* = 52	Increase Socio- demographic Parameters *n* = 87	Kruskal–Wallis Test *p*	Decrease *n* = 20	No Change *n* = 42	Increase *n* = 46	Kruskal–Wallis Test *p*
	%—Percentage of Respondents				%—Percentage of Respondents			
Before the SARS-CoV-2 pandemic (%)								
<2 h	16.3	19.2	19.5	0.3149	25.0	14.3	10.9	0.8445
2 to 4 h	18.6	25.0	21.8		15.0	23.8	19.6	
4 to 6 h	14.0	17.3	23.0		10.0	16.7	17.4	
6 to 8 h	20.9	17.3	17.2		5.0	16.7	19.6	
8 to 10 h	18.6	19.2	12.6		15.0	14.3	23.9	
>10 h	11.6	1.9	5.7		30.0	14.3	8.7	
During the SARS-CoV-2 pandemic (%)								
<2 h	16.3	9.6	9.2	0.1377	20.0	11.9	6.5	0.4950
2 to 4 h	14.0	21.2	13.8		5.0	11.9	10.9	
4 to 6 h	25.6	21.2	12.6		25.0	11.9	15.2	
6 to 8 h	11.6	23.1	21.8		5.0	28.6	13.0	
8 to 10 h	9.3	17.3	26.4		10.0	16.7	28.3	
>10 h	23.3	7.7	16.1		35.0	19.0	26.1	

## Data Availability

All results are available from the authors.

## Supplementary Materials

The following supporting information can be downloaded at: https://www.mdpi.com/article/10.3390/nu15112427/s1, Figure S1: Changes in form in women (a) and men (b) in subgroups with a decrease, no change, or increase in BMI before and during isolation; Figure S2: Change in physical activity while working in groups with decrease in BMI (a), and no change in BMI (b); Figure S3: Change in leisure-time physical activity in groups with decrease in BMI (a), and no change in BMI (b); Table S1: Frequency of eating out in subgroups of men and women in which there was a decrease, no change, or increase in BMI before and during isolation.
