# Supplementary material for: Lifestyle Changes during the SARS-CoV-2 Pandemic as Predictors of BMI Changes among Men and Women in Poland
Source: Nutrients. 2023 May 23;15(11):2427. doi: 10.3390/nu15112427 (PMC10254752; doi:10.3390/nu15112427)
Supplement: Supplementary file 1 [file nutrients-15-02427-s001.zip › nutrients-2368515-supplementary.pdf]

Suplement

a)

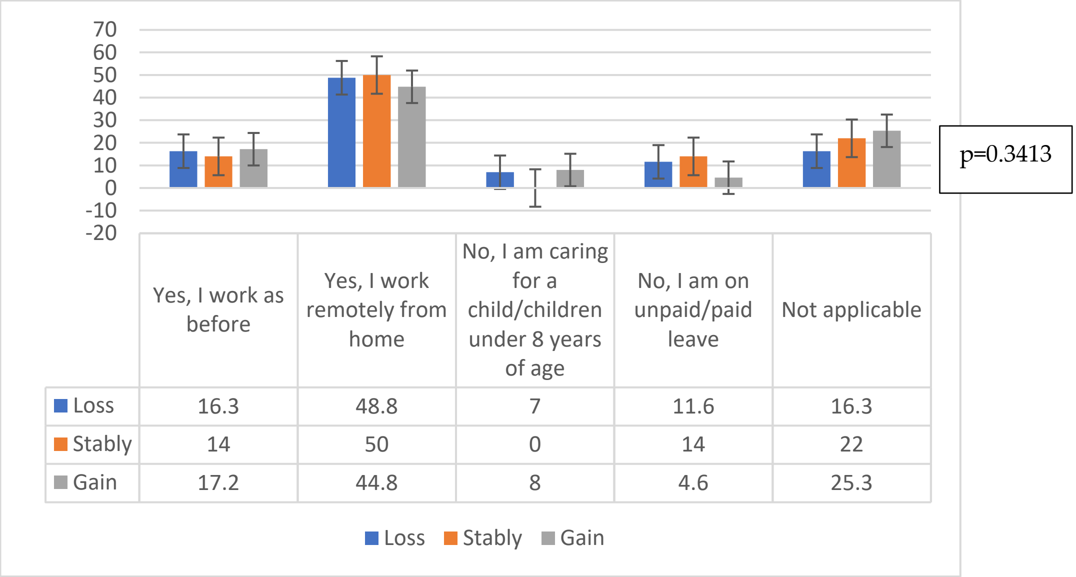

b)

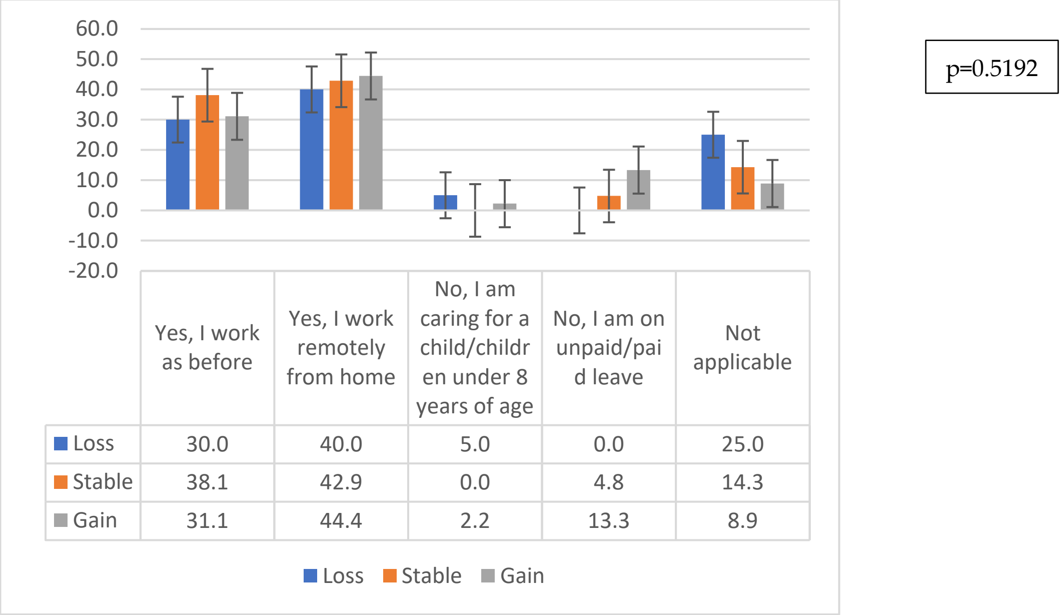

Figure S1. Changes in form in women a) and men b) in subgroups with a decrease, no change or increase in BMI before and during isolation.

Table S1. Frequency of eating out in subgroups of men and women in which there was a decrease no change or increase in BMI before and during isolation.

Variables	**Frequency of meals away from home (%)**							
	**Women**				**Men**			
	Loss<br>n = 43	Stable<br>n = 52	Gain<br>n = 87	p	Loss<br>n = 20	Stable<br>n = 42	Gain<br>n = 46	p

Before isolation caused by SARS-COV-2								
Never	9.3	19.2	17.2		15.0	19.0	13.0	
1-3 times a month	44.2	51.9	48.3		35.0	54.8	26.1	
Once a week	20.9	17.3	20.7	0.1137	15.0	7.10	23.9	0.0454
Several times a week	25.6	7.70	10.3		30.0	11.9	30.4	
Once a day	0.00	3.8	2.30		5.00	7.10	4.30	
Several times a day	0.00	0.00	1.10		0.00	0.00	2.20	
During the isolation caused by SARS-COV-2								
Never	46.5	55.8	58.6		55.0	52.4	37.0	
1-3 times a month	37.2	34.6	36.8		15.0	31.0	37.0	
Once a week	11.6	7.7	2.30	0.2360	20.0	14.3	13.0	0.2636
Several times a week	2.30	1.90	2.30		5.00	2.40	10.9	
Once a day	2.30	0.00	0.00		5.00	0.00	2.20	

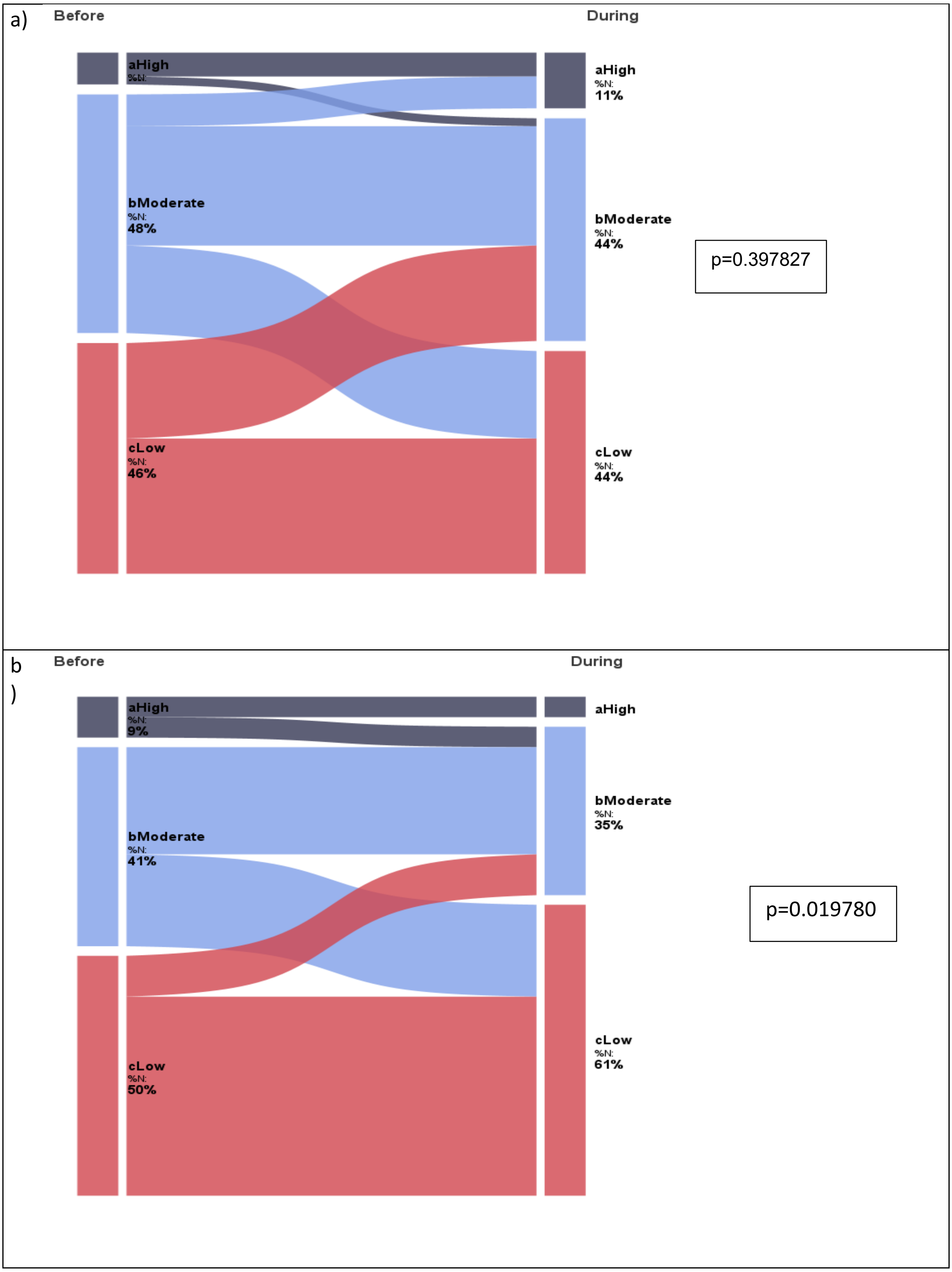

Question on physical activity during work or school time, with three possible levels with scores – c- low: more than 70% of the time is sitting - 1; b - moderate: about 50% of the time is sitting and 50% activity - 2; c - high: about 70% of the time is high-intensity activity or physical work - 3; and in leisure time, with responses and scores respectively - low: mainly sedentary lifestyle.

Figure S2. Change in physical activity while working in groups with decrease in BMI a), no change in BMI b).

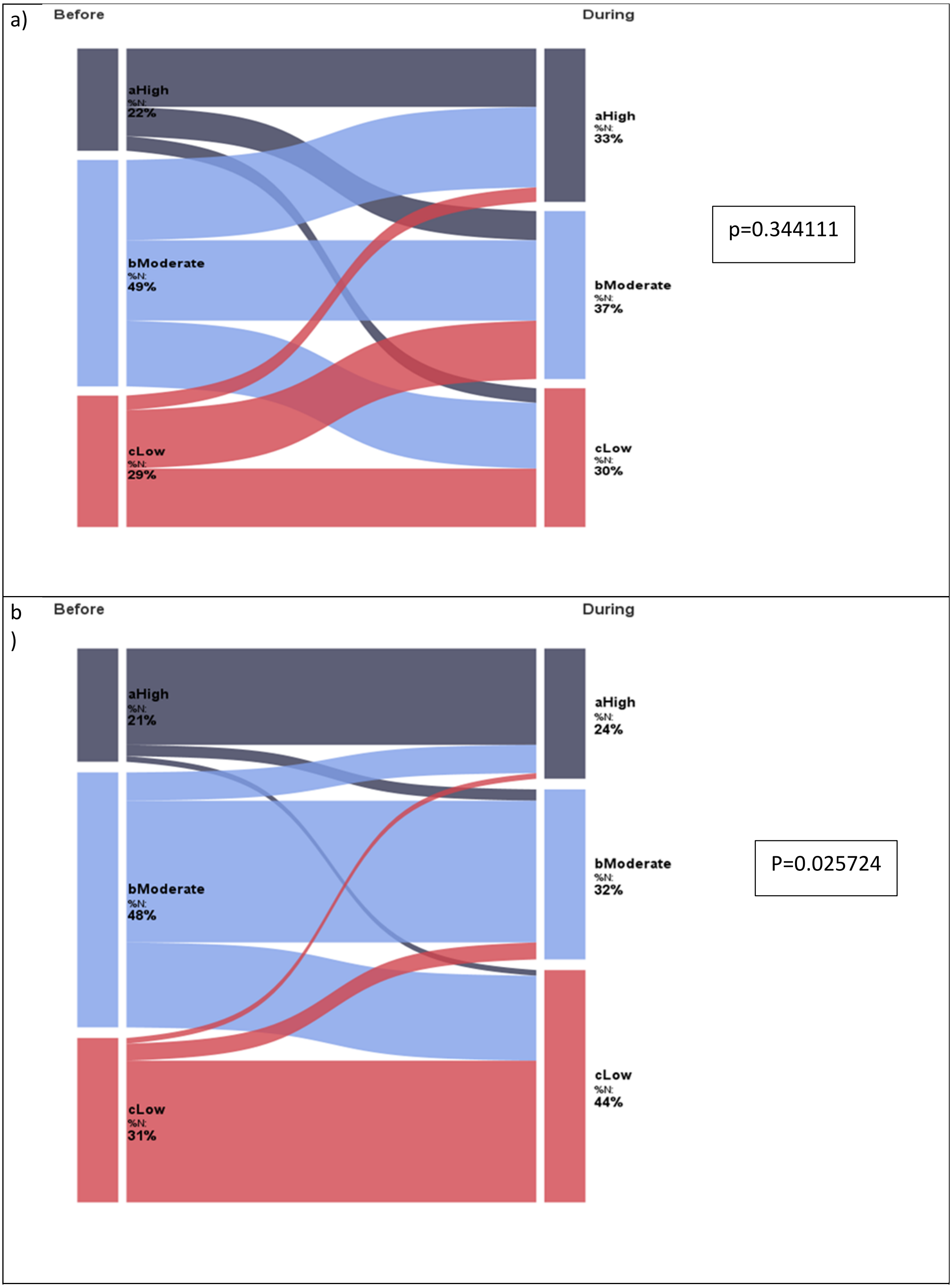

Question on physical activity during work or school time, with three possible levels with scores – c- low: more than 70% of the time is sitting - 1; b - moderate: about 50% of the time is sitting and 50% activity - 2; c - high: about 70% of the time is high-intensity activity or physical work - 3; and in leisure time, with responses and scores respectively - low: mainly sedentary lifestyle.

Figure S3. Change in leisure-time physical activity in groups with decrease in BMI a), no change in BMI b).
